# Next-generation CDK inhibitors in HR^+^/HER2^–^ breast cancer

**DOI:** 10.1172/JCI207631

**Published:** 2026-10-15

**Authors:** Daria Seriak, Jun Yong Kim, Geoffrey I. Shapiro, Antonio Giordano

**Affiliations:** 1 Dana-Farber Cancer Institute at Boston Medical Center, Brighton, Massachusetts, USA.; 2 Department of Medicine, Boston University Chobanian & Avedisian School of Medicine, Boston, Massachusetts, USA.; 3 Department of Medical Oncology, Dana-Farber Cancer Institute, Boston, Massachusetts, USA.; 4 Department of Medicine, Brigham and Women’s Hospital and Harvard Medical, School, Boston, Massachusetts, USA.

## Abstract

Cell-cycle dysregulation is a central driver of breast cancer progression and a major therapeutic vulnerability, particularly in hormone receptor–positive/human epidermal growth factor receptor 2–negative (HR^+^/HER2^–^) disease. The clinical success of cyclin-dependent kinase (CDK) 4/6 inhibitors combined with endocrine therapy has transformed treatment paradigms and established cell-cycle targeting as a cornerstone of care. However, important challenges remain, including hematologic toxicity, incomplete and largely cytostatic responses, and the near-universal development of resistance. These limitations have highlighted the need for next-generation CDK-directed strategies capable of improving selectivity, extending disease control, and overcoming adaptive escape pathways. This Review examines the evolving landscape of CDK-targeted therapeutics in breast cancer, with particular emphasis on emerging CDK4-selective inhibitors, CDK2-directed approaches, and broader multi-CDK strategies. We discuss the biological rationale underpinning these agents, the mechanisms of resistance that have informed their development, and the clinical opportunities they may create across both advanced and early-stage settings. By refining target specificity and addressing resistance-associated cell-cycle rewiring, next-generation CDK inhibitors may reduce toxicity, enhance treatment durability, and enable more effective combination and sequencing strategies. Collectively, these advances promise to move breast cancer treatment beyond current CDK4/6 inhibition toward more precise, adaptable, and durable cell-cycle–directed therapy.

## Introduction

Dysregulated control of the cell cycle is a fundamental hallmark of malignancy that enables sustained proliferative signaling across many tumor types, including breast cancer (1). Cyclin-dependent kinases (CDKs) are serine/threonine kinases that, together with their cyclin partners, coordinate orderly progression through the G_1_, S, G_2_, and M phases of the cell cycle, integrating mitogenic signals, checkpoint control, and DNA damage responses (2, 3). The 20 human CDKs include cell-cycle, transcriptional, and atypical kinases (3). Upon mitogenic stimulation, cyclin D–CDK4/6 complexes initiate phosphorylation of the retinoblastoma (Rb) tumor suppressor protein in early G_1_, generating monophosphorylated Rb isoforms that partially relieve repression of E2F transcription factors (E2Fs) (4, 5). Complete Rb inactivation, however, has been thought to require subsequent hyperphosphorylation by cyclin E–CDK2 complexes in late G_1_, promoting S phase entry (6–8). Single-cell studies have refined this model, showing that partial Rb phosphorylation by either CDK4/6 or CDK2 can generate a reversible intermediate state before full cyclin E–CDK2–dependent commitment to S phase entry (9).

In breast epithelium, estrogen receptor (ER) signaling directly upregulates cyclin D1 (*CCND1*) gene expression, creating a positive feedback loop in which cyclin D1–CDK4/6 complexes reciprocally enhance ER transcriptional activity (10, 11). Additionally, oncogenic alterations associated with breast cancer frequently converge on the cyclin D–CDK4/6–Rb axis. Amplification or overexpression of *CCND1*, activation of upstream growth factor signaling (e.g., PI3K/AKT/mTOR, FGFR, or MAPK pathways), and loss of negative regulators (such as p16INK4A) promote persistent CDK4/6 activity, leading to sustained Rb phosphorylation, activation of E2Fs, and, ultimately, the G_1_ to S transition (12) (Figure 1). Hormone receptor–positive/human epidermal growth factor receptor 2–negative (HR^+^/HER2^–^) breast cancer, which accounts for approximately 60%–70% of all breast cancers, is particularly dependent on this pathway, explaining the success of CDK4/6 inhibition in this disease (13).

Early pan-CDK inhibitors were limited by narrow therapeutic indices and dose-limiting toxicities (DLTs) and did not progress to clinical practice (14, 15). Improved structural comprehension of CDK-cyclin complexes and the advancement of more selective ATP-competitive inhibitors enabled development of more selective agents — palbociclib, ribociclib, and abemaciclib — which, in combination with endocrine therapy (ET), have transformed the management of HR^+^/HER2^–^ breast cancer, demonstrating consistent improvements in progression-free survival (PFS) and, for several regimens, overall survival, across metastatic and high-risk early-stage settings (16, 17). CDK4/6 inhibitors (CDK4/6is) plus ET are now the standard of care for most patients with HR^+^/HER2^–^ advanced breast cancer.

Despite these advances, important challenges motivate the development of next-generation CDK-directed therapies. Neutropenia and other hematologic toxicities frequently require dose interruptions or reductions, driving interest in CDK4-selective inhibitors (CDK4is) that limit myelosuppression (18). One important challenge is intrinsic (primary) resistance, in which tumors fail to respond at treatment initiation; this occurs in approximately 10%–20% of cases and likely reflects preexisting molecular or cell-state features that sustain proliferation despite CDK4/6 inhibition (19–21). In contrast, acquired resistance or adaptive escape develops in tumors that initially respond but later resume growth under treatment, often through bypass of CDK4/6 dependence and increased reliance on CDK2-related signaling (20, 22). Intrinsic heterogeneity in cell-cycle regulator expression also contributes to fractional resistance, in which a small subpopulation of cells within an otherwise CDK4/6i-sensitive tumor continues to proliferate due to cell-to-cell variability in regulators such as E2F1, Rb, and CDK2 (23). Finally, current CDK4/6is primarily induce a reversible cytostasis or senescence rather than cell death, and recovery of proliferative capacity following treatment interruption has been observed in several tumor contexts (24, 25). Together, these limitations underscore the need for agents that achieve more durable cell-cycle control, address both intrinsic and acquired resistance mechanisms, and support rational combination strategies without added toxicity.

With this growing interest, several recent reviews have addressed CDK-directed therapeutics in oncology (26, 27), but key distinctions motivate the present work. This Review focuses specifically on the next generation of CDK inhibitors in HR^+^/HER2^−^ breast cancer. We review CDK4is, CDK2-selective inhibitors (CDK2is), multitargeted CDK agents, and CDK-targeting proteolysis-targeting chimeras (PROTACs), with emphasis on resistance biology, biomarkers, as well as emerging combinations with novel ETs.

## CDK4is

CDK4 and CDK6 share approximately 70% sequence homology and jointly regulate Rb phosphorylation in early G_1_. However, CDK4 is the dominant driver of proliferation in many HR^+^/HER2^–^ breast cancers, while CDK6 primarily regulates proliferation and differentiation of hematopoietic stem and progenitor cells (3, 28). Preclinical work suggests that tumor dependence may be preferentially CDK4 or CDK6 driven (29), with HR^+^ breast cancer among the tumor types most dependent on CDK4 (30). Because current dual CDK4/6is poorly distinguish the highly similar ATP-binding pockets of CDK4 and CDK6 (31, 32), myelosuppression, particularly neutropenia, remains a major toxicity, attributable in part to CDK6 inhibition resulting in discontinuous and/or attenuated dosing (33, 34). Accordingly, the goal of CDK4i development is to reduce hematologic toxicity while preserving or intensifying antitumor efficacy (Table 1).

### Atirmociclib.

Atirmociclib (Pfizer Inc., previously known as PF-07220060) is a first-in-class, CDK4i that displays over 20-fold greater selectivity for CDK4 over CDK6 compared with palbociclib and approximately 3- to 5-fold greater selectivity compared with abemaciclib and ribociclib (30, 32). Preclinically, selective CDK4 inhibition reduced hematologic toxicity and enabled deeper suppression of Rb phosphorylation and tumor growth than palbociclib in CDK4-driven models, including ER^+^ breast cancer (30).

In the first-in-human phase I/IIa trial (monotherapy followed by combination with ET in HR^+^/HER2^–^ metastatic breast cancer (mBC), including patients progressing after prior CDK4/6is), atirmociclib showed a favorable tolerability profile and meaningful clinical activity (35). Pharmacodynamic analyses showed decreases in tumor Rb phosphorylation and Ki-67 expression as well as reduced serum TK1 activity and circulating tumor DNA (ctDNA). Based on the totality of data, 300 mg twice daily was selected for continuous dosing (35).

Among 34 patients treated in a first-line setting with atirmociclib (300 mg BID) plus letrozole, the most common treatment-related adverse events (TRAEs) were neutropenia (61.8% all grades; 26.5% grade 3) and leukopenia (41.2%; no grade 3); no diarrhea was reported (36). Dose intensity was largely maintained. Antitumor activity was also observed, with a confirmed objective response rate (ORR) of 69.7% and a clinical benefit rate (CBR) of 93.9%. After a median follow-up of 24.8 months (95% CI, 22.0–25.8 months), median PFS had not been reached; the estimated event-free probability was 90.9% at 12 months, 78.4% at 18 months, and 67.2% at 24 months (36).

Building upon these findings, a pivotal trial (FOURLIGHT-3, NCT06760637) was launched in January 2025 to investigate atirmociclib in the first-line setting. FOURLIGHT-3 is an open-label, randomized, multicenter phase III study comparing atirmociclib plus letrozole with standard-of-care CDK4/6is (palbociclib, ribociclib, or abemaciclib) plus letrozole in adults with previously untreated HR^+^/HER2^−^ advanced or mBC (37).

Atirmociclib also showed activity in patients previously exposed to CDK4/6is. Among patients with measurable disease who progressed on a prior CDK4/6i plus ET, atirmociclib plus letrozole or fulvestrant demonstrated a confirmed ORR of 32.0% (8/25), with 1 CR and 7 partial responses (PRs) (38). CBR was 60.6% (20/33). Median PFS was 8.1 months (95% CI: 5.3, 10.9 months). Responses occurred irrespective of *ESR1* or PI3K pathway mutations (38), suggesting preserved activity across common endocrine-resistance genotypes.

Based on activity observed in patients already exposed to CDK4/6is, FOURLIGHT-1 (NCT06105632) was initiated in September 2024 as an international randomized phase II trial comparing atirmociclib plus fulvestrant against investigator’s choice of fulvestrant alone or everolimus plus exemestane in patients who progressed on prior CDK4/6i therapy. According to a preliminary communication from Pfizer in March 2026, the trial met its primary endpoint, with a statistically significant improvement in investigator-assessed PFS (HR 0.60; 95% CI. 0.44–0.83; *P* = 0.0007) (39). A presurgical window study has also completed enrollment (NCT06465368). Owing to its favorable toxicity profile, atirmociclib has been the partner of choice in a triplet-combination trial incorporating the mutant-selective PI3Kα inhibitor zovegalisib with CDK4 inhibition plus ET (NCT05216432) and in combinations with camizestrant, a next-generation selective ER degrader (SERD; NCT07427394), and vepdegestrant, a PROTAC ER degrader (NCT06206837).

Combining atirmociclib with novel agents, such as oral SERDs or PROTAC-based ER degraders, represents a particularly promising therapeutic strategy. The TACTIVE-K study (NCT06206837) investigates atirmociclib with vepdegestrant in post-CDK4/6i ER^+^/HER2^−^ disease. Preliminary phase Ib/II data reported an ORR of 16%–24% and median PFS of 9.3–11.5 months across dose cohorts, with consistent activity in patients harboring *ESR1* mutations and a safety profile broadly consistent with each agent alone (40). Another trial (NCT05216432) is investigating the mutant-selective PI3Kα inhibitor zovegalisib (RLY-2608, Relay Therapeutics) in triplet combinations with fulvestrant and either a CDK4/6i or atirmociclib in PIK3CA-mutated HR^+^/HER2^−^ breast cancer. Preliminary data from the zovegalisib plus atirmociclib plus fulvestrant cohort, reported in an unpublished company press release in April 2026, demonstrated a 44% ORR in heavily pretreated patients (patients are on a median of their third-line treatment, and all are CDK4/6i experienced) (41). Tolerability appeared consistent with individual agents, and a phase III frontline trial is planned pending regulatory feedback.

Clinical trials will ultimately assess intrinsic resistance as well as the emergence of tumors with acquired resistance to atirmociclib. To date, preliminary ctDNA analyses suggest that acquired resistance mechanisms may at least partially overlap those of dual CDK4/6is (42). Consistent with this possibility, in the initial preclinical description of atirmociclib in an ER^+^/HER2^–^ breast cancer xenograft model, activation of the cyclin E1–CDK2 complex and FGFR1 signaling were both associated with acquired resistance (30). Additionally, CDK6 may be particularly relevant to resistance in the setting of selective CDK4 inhibition. Baseline CDK6 expression mediated intrinsic resistance to atirmociclib in preclinical models, and genomic alterations such as *FAT1*, *PTEN,* and *ARID1A* loss may converge on CDK6 upregulation in tumors less likely to benefit from CDK4-selective inhibition (30, 43, 44). Notably, CDK6 overexpression has also been implicated in acquired resistance to dual CDK4/6is (45, 46). Inhibition of bromodomain and extra-terminal (BET) proteins can suppress CDK6 expression in resistant models, suggesting a potential combination strategy (47).

### Additional CDK4-selective agents.

BGB-43395 (BeOne Medicines) is another next-generation, orally administered CDK4i. Preclinically, BGB-43395 showed potent and selective CDK4 inhibition with strong suppression of Rb phosphorylation and tumor growth in HR^+^/HER2^−^ models (48). Preliminary clinical data from the first-in-human phase Ia trial (NCT06120283) indicated that BGB-43395 was tolerable in early dose-escalation and combination cohorts (49). A first-line safety expansion cohort evaluated BGB-43395 plus letrozole in 58 CDK4/6i-naive patients with HR^+^/HER2^−^ advanced or mBC across three doses (240, 400, and 600 mg BID). As of April 30, 2026, toxicity was manageable at 240 and 400 mg BID. Most adverse events were grade 1–2 gastrointestinal events with low hematologic toxicity and no TRAE led to discontinuation at the recommended phase III dose of 400 mg BID. Confirmed ORRs ranged from 55.0% to 68.4% across dose levels (50). A first-line phase III trial has been launched (NCT07492641).

Several other CDK4-selective compounds have recently entered early-phase clinical evaluation, including HRS-6209 and AVZO-023 (Table 1). In summary, the promising outlook of CDK4is supports the central role of CDK4 in driving proliferation in HR^+^/HER2^–^ breast cancer and suggests that selective CDK4 targeting may preserve or even enhance antitumor efficacy while decreasing hematologic toxicity mediated by CDK6 inhibition.

## CDK2is

### Rationale for selective CDK2 inhibition.

Despite the success of CDK4/6is plus ET in HR^+^/HER2^–^ breast cancer, resistance to these agents is almost universal (19–21). Many resistance mechanisms converge on elevated CDK2 activity, including cyclin E1 overexpression, *CCNE1* or *CCNE2* amplification, noncanonical cyclin D1–CDK2 activation, and upstream activation of PI3K–AKT–mTOR, FGFR, or MAPK signaling, all of which can restore Rb phosphorylation and cell-cycle progression despite CDK4/6 inhibition (20, 21, 51–53). In addition to CDK4/6i-resistant HR^+^/HER2^–^ breast cancer, *CCNE1* amplification occurs in subsets of other tumor types, including triple-negative breast cancer (TNBC) (54), high-grade serous ovarian cancer (HGSOC) (55), and upper GI malignancies (56), contributing to the rationale for developing CDK2is.

### Biomarkers of response to CDK2 inhibition.

Although patients with CDK4/6i-resistant HR^+^/HER2^–^ breast cancers or other tumors with cyclin E1 overexpression or *CCNE1* or *CCNE2* amplification comprise rational populations for selective CDK2i trials, it is important to note that CDK2 inhibition can induce two mechanistically distinct cell-cycle arrests at either G_1_ or G_2_, depending on the tumor genetic makeup (22, 57). This distinction is important for patient selection. In *RB1*-intact tumors with high cyclin E that retain p16INK4A expression, as is the case in subsets of TNBC or gynecologic cancers, CDK2 is the dominant driver of Rb phosphorylation. A high cyclin E level renders cancer cells dependent on CDK2, while a high p16INK4A level blocks CDK4/6, further augmenting dependence on the cyclin E–CDK2 complex. In this context, CDK2 inhibition induces G_1_ arrest and antiproliferative effects (57–60). Indeed, emerging data indicate that high levels of cyclin E and p16INK4A expression are promising biomarkers of response. In models of HGSOC and endometrial cancer, *CCNE1*-amplified tumors with intact Rb and high p16INK4A expression exhibited potent sensitivity to CDK2i inhibition, including BLU-222 monotherapy (59, 60). Another CDK2i, INX-315, also inhibited tumor growth with greater potency in tumors expressing both cyclin E1 and p16INK4A (57).

In contrast, *RB1*-intact tumors carrying *CDKN2A* deletion, mutation, or promoter methylation are CDK4- or CDK4/6-driven, as occurs in most HR^+^/HER2^−^ breast cancers. Here, CDK2 inhibition alone does not disrupt G_1_/S progression (61). Instead, cells are arrested at G_2_ resulting from inhibition of cyclin A–CDK2–mediated phosphorylation of substrates such as B-MYB and FOXM1 (58, 62, 63), events required for G_2_/M progression. However, cell-cycle arrest is partial and easily overcome without concomitant CDK1 inhibition (64), suggesting that, in this setting, selective CDK2i monotherapy will have modest activity. Rb-deficient tumors may similarly undergo G_2_ arrest upon CDK2 inhibition (57) and may also be prone to apoptosis resulting from persistently high E2F1 activity (65), suggesting *RB1* alteration as a possible biomarker.

In CDK4/6i-resistant HR^+^/HER2^−^ breast cancer, convergent resistance mechanisms position CDK2 as a central downstream node sustaining proliferation after CDK4/6i failure, making it an attractive target to address multiple resistance drivers (20). Prior CDK4/6i exposure may shift tumors toward greater reliance on CDK2, potentially increasing sensitivity to CDK2 inhibition, so that monotherapy may benefit a subset of patients with acquired CDK4/6i resistance. However, such tumors may still be p16INK4A-deficient. Indeed, preclinical studies indicate that continued CDK4/6 inhibition with the cotargeting of CDK2 suppressed the growth of endocrine- and CDK4/6i–resistant ER^+^ breast cancer models, reestablishing cell-cycle arrest, and delayed tumor progression in resistant xenograft settings (66, 67). A novel, weighted mRNA expression signature involving *CCND1*, *CCNE1*, *RB1*, and *CDKN2A* (p16INK4A) has been shown to predict response to combined inhibition of CDK2 and CDK4/6 in HR^+^/HER2^–^ (CDK4/6i naive and resistant) and TNBC models (68). Collectively, these biological considerations and preclinical findings provide a clear rationale for developing CDK2is (Table 2) as well as insight into emerging monotherapy clinical data.

### Tegtociclib.

Tegtociclib, also known as PF-07104091 (Pfizer Inc.), is a first-in-class CDK2i. After showing preclinical activity in xenograft models, including PDX HR^+^ breast cancer models with acquired resistance to CDK4/6is and ET (69), tegtociclib has been investigated in a phase I/IIa study in patients with advanced or metastatic solid tumors, including a heavily pretreated cohort with HR^+^/HER2^−^ mBC who had progressed on prior ET and CDK4/6is (NCT04553133). Preliminary results indicate that tegtociclib has been tolerated at escalating dose levels, with gastrointestinal toxicities and fatigue as the most common adverse events. Pharmacokinetics (PK) analyses revealed dose-proportional exposure associated with early ctDNA decrease, with the maximum tolerated dose (MTD) defined at 300 mg twice daily (70). Among 16 response-evaluable heavily pretreated patients with mBC, all of whom had received a prior CDK4/6i, confirmed PRs were observed in 3 patients (18.8%, with duration of response >6 months in 2 patients and 1 ongoing at the time of reporting), and stable disease (SD) was observed in 6 patients (37.5%), with a disease control rate of 61.5% (95% CI, 40.6%–79.8%).

These data suggest that tegtociclib as monotherapy has meaningful activity in a subset of patients with HR^+^/HER2^−^ breast cancer with acquired CDK4/6i resistance, and the results were confirmed in a recently reported cohort of 10 additional patients, in which there was 1 PR and 4 instances of SD (71). Notably, clinical benefit, manifested by duration of treatment between 93 and 266 days, was observed irrespective of Rb expression and occurred only in patients whose tumors were *TP53* wild-type. Surprisingly, studies in patient-derived models demonstrated that tegtociclib reduced the DNA replication rate without reduction of Rb phosphorylation (70). Instead, tegtociclib inhibited mitotic entry, with growth inhibition dependent on P53 expression, and tegtociclib induced DNA damage as well. These findings support a shift in CDK dependency from CDK4/6 toward CDK2 as cells transition from a CDK4/6i-sensitive to a CDK4/6i-resistant state, with effects of CDK2 inhibition and CDK4/6 inhibition targeting distinct cell-cycle phases.

### BLU-222.

BLU-222 (Blueprint Medicines) is an oral, potent, and highly selective CDK2i. Preclinical studies demonstrated robust antitumor activity of BLU-222 in HR^+^/HER2^−^ breast cancer models. Cyclin E1–driven ribociclib-resistant models that retained p16INK4A expression regressed with BLU-222 monotherapy (72). Clinical evaluation of BLU-222 was clinically evaluated in the phase I/II VELA trial (NCT05252416), which enrolled heavily pretreated patients (median 5 prior lines) with breast, ovarian, endometrial, and prostate cancer. At data cutoff, the MTD had not been reached. Common toxicities included gastrointestinal events, fatigue, anemia, photophobia, and hypokalemia, with preliminary activity observed before the program was discontinued for strategic reasons (73).

### INCB123667.

INCB123667 (Incyte) is a potent, selective CDK2i investigated in a phase I, open-label, dose-escalation and dose-expansion study (NCT05238922). The study enrolled patients with advanced/metastatic solid tumors and measurable disease, with *CCNE1* amplification preferred during dose escalation and required in expansion cohorts (74). Among 224 treated patients, the safety profile was dominated by hematologic and gastrointestinal toxicities; the 50 mg BID schedule showed the most favorable gastrointestinal tolerability and was selected for further development. Notably, in the ovarian cancer expansion cohort (*CCNE1*-selected), an ORR of 33.3% and a median PFS of 5.3 months were observed (74).

### INX-315.

INX-315 (Incyclix Bio) is a potent and highly selective CDK2i. Preclinically, INX-315 showed marked activity in *CCNE1*-amplified tumors and in CDK4/6i-resistant breast cancer models with upregulated *CCNE1*, supporting CDK2 monotherapy in selected tumors (58). INX-315 is now being clinically investigated in a phase I/II trial in both *CCNE1*-amplified cancers and CDK4/6i-resistant breast cancer (NCT05735080). As of June 2024, 27 patients were enrolled across 5 dose cohorts and included in the safety analysis, with a heavily pretreated population (median 5 prior lines) comprising ER^+^/HER2^−^ breast cancer (*n* = 8), ovarian cancer (*n* = 10), and other solid tumors (*n* = 9) (75). Among efficacy-evaluable patients, 2 confirmed PRs were observed (1 ER^+^/HER2^−^ breast cancer, 1 *CCNE1*-amplified ovarian cancer), and 14 patients (63.6%) achieved SD. Plasma concentrations achieved effective trough levels at the lowest dose, supporting once-daily dosing (75).

### BG-68501.

BG-68501 (BeOne Medicines) is a highly potent, selective CDK2i that induces novel interactions within the CDK2 ATP-binding pocket, resulting in tight binding with a slow off-rate. It has >100-fold selectivity for CDK2 and has shown preclinical activity in *CCNE1*-amplified cancers, Rb-deficient small cell lung cancer, and CDK4/6i-resistant breast cancer models (76). BG-68501 is currently in a first-in-human phase Ia/Ib clinical trial (NCT06257264) in patients with advanced solid tumors, including HR^+^/HER2^−^ breast cancer (all with prior CDK4/6i therapy). At a data cutoff, TEAEs occurred in 95.1% of patients (grade ≥3, 26.8%) (77). Gastrointestinal toxicity predominated, although no DLTs were observed; notably, myelosuppression was not a prominent feature. Among 24 efficacy-evaluable patients, 1 heavily pretreated HR^+^/HER2^−^ breast cancer patient achieved a confirmed PR, and 10 achieved SD (77). Dose escalation for both monotherapy and the fulvestrant combination is ongoing.Several other CDK2is have entered phase I clinical trials: AVZO-021, AZD8421, ECI830, and NKT3447 (Table 2). In summary, as monotherapies, these agents have been tolerable, albeit with gastrointestinal toxicities, and may benefit a subset of biomarker-selected patients with HR^+^/HER2^–^ breast cancer with acquired resistance to CDK4/6 inhibition.

## Combining CDK2 and CDK4/6 or CDK4-selective inhibition

In CDK4/6i-naive breast cancer cells (i.e., the CDK4/6i-sensitive state), combined CDK2 and CDK4/6 inhibition may more completely suppress Rb phosphorylation and limit adaptive cell-cycle rewiring. However, such combinations may have greatest impact in CDK4/6i-resistant cells. Although CDK2i monotherapy studies have demonstrated benefit in patients with CDK4/6i resistance, multiple preclinical studies have supported the simultaneous inhibition of CDK2 and CDK4/6 as a potentially more effective strategy to overcome resistance (58, 66–69, 72, 78, 79). The findings of shifted dependence to CDK2 in CDK4/6i-resistant cells, and that CDK2 inhibition can suppress mitotic entry independent of Rb expression with induction of DNA damage, suggest distinct mechanisms of CDK2 and CDK4/6 inhibition and explain why synergism is enhanced in resistant models (58, 60, 69, 71, 72, 78–80). Continued CDK4/6 inhibition contributes to growth arrest in p16INK4A-low cells, whereas CDK2 inhibition effectively targets cells that have escaped the G_1_/S checkpoint. Because CDK6 inhibition contributes to neutropenia, pairing a CDK2i with a CDK4-selective agent may also improve tolerability. In a phase I/II clinical trial (NCT05262400), the combination of atirmociclib and tegtociclib demonstrated a 27.8% PR rate and 27.8% SD rate among patients with mBC with measurable disease (*n* = 18), and a 69.2% disease control rate with a median PFS of 8.3 months among the broader mBC cohort (*n* = 26) in heavily pretreated, CDK4/6i-exposed mBC (81).

## Multi-CDK inhibitors

In addition to combination strategies, novel compounds have been engineered to simultaneously inhibit CDK2 and CDK4 within a single molecule (Table 3). PF-06873600 (Pfizer), a first-in-class CDK2/4/6i, demonstrated preliminary antitumor activity in a phase I/IIa trial (NCT03519178), including an ORR of 22.7% and a median PFS of 11.1 months in CDK4/6i-naive patients (82). However, its narrow therapeutic index and limited target coverage led to discontinuation in favor of more selective next-generation agents.

In contrast, GDC-4198 (formerly RGT-419B, Genentech/Regor Therapeutics) is a novel CDK4/2i with subnanomolar potency against CDK4 and greater activity against CDK2 than CDK6, designed to overcome resistance to existing CDK4/6is and reduce hematologic toxicity. Preclinically, it induced durable cell-cycle arrest and inhibited tumor growth more potently than CDK4/6is in resistant HR^+^ breast cancer models (83). In a first-in-human phase I trial, patients with heavily pretreated HR^+^/HER2^−^ mBC (median 3 prior lines, 100% prior CDK4/6i) received GDC-4198 as monotherapy (25–150 mg QD or 150 mg BID) or in combination with an aromatase inhibitor. The drug was well tolerated; the most common TRAEs were nausea, diarrhea, vomiting, and hematologic changes, with a low rate of grade ≥3 TRAEs (16%) and no treatment-related discontinuations (84). The MTD had not been reached at the time of reporting. Among 24 patients with measurable disease and at least 1 postbaseline assessment, 4 confirmed PRs were observed in the monotherapy cohorts (1 at 150 mg QD, 3 at 150 mg BID). Dose escalation is ongoing, as well as additional studies in combination with giredestrant and other ET (NCT07100106, NCT05304962).

## CDK-targeting PROTACs

PROTACs are heterobifunctional molecules that recruit an E3 ubiquitin ligase to a target protein, inducing its ubiquitination and proteasomal degradation (85). CDK-targeting PROTACs offer several key advantages over classical small-molecule inhibitors. By eliminating the target protein, they can suppress both kinase-dependent and kinase-independent CDK functions and may achieve selectivity not attainable with catalytic inhibitors (44, 86, 87). Key challenges include high molecular weight, limited oral bioavailability, and dependence on E3 ligase expression. Most CDK PROTACs currently rely on cereblon or VHL E3 ligases, and resistance through ligase downregulation has already been anticipated (87, 88). Most CDK degraders utilize a warhead targeting the active kinase derived from ATP-competitive inhibitors (i.e., type I degraders) (Table 3).

### BTX-9341.

BTX-9341 is an orally bioavailable PROTAC degrader targeting CDK4/6 that showed preclinical antitumor activity in HR^+^ and *CCNE1*-high breast cancer models superior to conventional CDK4/6is, including in CDK4/6i-resistant settings (89). This agent has also been reported to cross the blood-brain barrier. In the first-in-human study of BTX-9341 (NCT06515470) in *RB1* wild-type HR^+^/HER2^−^ mBC, no DLTs occurred, on-target pharmacodynamic activity was confirmed, and early efficacy signals were observed, with 1 confirmed PR and a CBR of 66.7% at week 24 (90).

Palbociclib-based PROTAC probes capable of dual or paralog-selective CDK4/6 degradation have also been described (91). As CDK6 upregulation is an established driver of acquired CDK4/6i resistance in ER^+^ breast cancer, selective CDK6 degradation is a biologically justified strategy in this setting (43–46). CDK6-selective PROTACs are therefore being developed, although early compounds have been limited by suboptimal drug-like properties and pharmacokinetics (87, 88, 92–95). Additionally, degradation by these compounds depends on the presence of low INK4 protein levels (96).

Type I degraders have also demonstrated a hook effect, i.e., a bell-shaped dose-response curve, where CDK4/6 degradation drops at high concentrations, occurring when excess bifunctional degrader molecules saturate individual binding sites, forming separate, nonproductive binary complexes (CDK-degrader or E3-degrader) instead of the required productive ternary complex (CDK-degrader-E3 ligase). Recently, a type II CDK6 degrader has been described that promotes efficient CDK6 degradation despite weak CDK6 binding to the inactive conformation and negligible CDK6 inhibition, producing Rb dephosphorylation and sustained G_1_ arrest in CDK6-dependent models (97). Importantly, this compound did not display a hook effect. Whether low INK4 levels are required for efficient degradation with a type II CDK6 PROTAC remains to be tested.

In the CDK2 space, NKT3964 (NiKang Therapeutics) is a first-in-class, orally bioavailable CDK2 degrader that selectively degrades CDK2 with high specificity over other CDK family members, causing prolonged CDK2 pathway inhibition without cyclin E accumulation (98). Designed to treat patients with aberrant CDK2/cyclin E pathway activation, including those with HR^+^/HER2^−^ breast cancer following progression on a CDK4/6i, NKT3964 has advanced to a phase I study (NCT06586957). A second compound, NKT5097, is a highly selective CDK2/4 degrader, with activity in HR^+^/HER2^−^ breast cancer and cyclin E–high cancer models, and has also entered phase I investigation (NCT07029399) (Table 3) (99).

Notably, peer-reviewed clinical data for the CDK-targeting PROTACs discussed here remain unavailable. The recent FDA approval of vepdegestrant, the first PROTAC to reach regulatory approval in oncology (100, 101), nevertheless demonstrates that targeted protein degradation is clinically actionable and sets a precedent for CDK-directed programs.

## CDK7 inhibitors

Another CDK-directed strategy under investigation targets CDK7. CDK7 regulates both cell-cycle progression and transcriptional control as a component of the CDK-activating kinase (CAK) complex and RNA polymerase II C-terminal domain (CTD) kinase (102–104). The CAK complex is overexpressed in breast cancer, including ER^+^ breast cancer and TNBC (105, 106). As inhibition of CAK activity is expected to simultaneously reduce activating phosphorylation of both CDK4 and CDK2, selective CDK7 inhibition is an attractive strategy in CDK4/6i-resistant disease. CDK7 also phosphorylates ER at S118; although CDK7 inhibition suppresses this phosphorylation, long-term CDK7i treatment in preclinical models increased ER signaling, supporting continued ET with CDK7 inhibition. Furthermore, inhibition of CDK7-mediated transcriptional activity suppresses *MYC* expression and MYC signaling, also contributing to its effects in CDK4/6i-resistant disease and suggesting a useful strategy in TNBC (107). In a phase I trial, samuraciclib, an oral selective CDK7 inhibitor, showed a CBR of 36.0% (9/25) in combination with fulvestrant in post-CDK4/6i HR^+^/HER2^−^ breast cancer and 20.0% (4/20) as monotherapy in TNBC, with mostly low-grade gastrointestinal adverse events (108). Phase II trials with fulvestrant, elacestrant, and vepdegestrant are ongoing, with other CDK7 inhibitors also in development (NCT05963984, NCT05963997, NCT06125522, NCT04247126, NCT05394103).

## Discussion

The addition of CDK4/6 inhibition to ET has produced tremendous clinical success in HR^+^/HER2^−^ breast cancer but is inadequate for long-term disease control. Next-generation CDK-directed agents aim to address this limitation through deeper and more selective inhibition of the cell-cycle machinery.

Rb phosphorylation and S phase entry can be restored through *CCNE1* amplification, noncanonical cyclin D1–CDK2 complex activation, CDK6 overexpression, and upstream oncogenic signaling despite efficient CDK4/6 blockade. This plasticity, facilitated by functional redundancy among several CDK family members, is arguably the core challenge to achieving long-lasting cell-cycle arrest. This provides the rationale for combination strategies and multitargeting CDK inhibitors.

Initial pan-CDK inhibitors broadly inhibited multiple CDK family members without isoform selectivity, rendering them highly toxic and yielding only modest efficacy. The current class of agents represents a more tailored strategy: targeting specific kinases that drive tumor cell-cycle progression and mediate resistance, primarily CDK4 and CDK2, while deliberately sparing those whose inhibition contributes disproportionately to toxicity.

CDK4is are being developed to improve tolerability by sparing CDK6, which may reduce CDK6-associated hematologic toxicity, particularly neutropenia.

Among CDK4-selective agents, both atirmociclib and BGB-43395 are under evaluation in phase III trials in the first-line setting for HR^+^/HER2^−^ advanced breast cancer, with results pending. Atirmociclib in combination with ET has also demonstrated activity in breast cancers with acquired CDK4/6i resistance in a randomized phase I/II trial (NCT04557449) (35, 38). As CDK4/6i resistance mechanisms have not been associated with *CDK4* amplification or otherwise elevated CDK4 activity, these results were unanticipated. They may reflect tumor heterogeneity, with only a subset of tumor cells demonstrating emerging resistance and other cells still maintaining profound CDK4 dependence, remaining prone to the antiproliferative and/or prosenescence effects of potent selective CDK4 inhibition applied continuously. Additionally, not all CDK4/6i resistance mechanisms occur at the genomic level, and in some cases, gene expression changes or miRNA expression may mediate resistance, effects that may be reversible (46), so that reapplication of CDK4 inhibition after a brief time off the initial CDK4/6 agent may produce antitumor efficacy. Finally, these results raise the question of whether weak inhibitory activity of atirmociclib against other kinases identified in biochemical assays (30) could manifest in HR^+^ breast cancer cells, contributing to these results.

However, greater selectivity for CDK4 does not necessarily address common resistance mechanisms to CDK4/6 pathway inhibition, especially those that bypass Rb-dependent cell-cycle control through activation of the cyclin E–CDK2 axis. For CDK2is, early clinical data suggest activity in CDK4/6i-pretreated populations; however, several limitations remain. Most trials are performed in heavily pretreated, biomarker-unselected, or heterogeneously selected cohorts, making cross-trial comparisons unreliable. Prospective validation of the optimal biomarker strategy (e.g., *CCNE1* amplification, p16INK4A loss, or composite CDK2 dependency signatures) is warranted. Furthermore, the gastrointestinal toxicities and myelosuppression seen with several CDK2is, while generally manageable, raise questions over combination tolerability with CDK4is. The lack of significant myelosuppression observed in BG-68501–treated patients suggests that kinetic and structural differences among CDK2is may translate into distinct toxicity profiles. Finally, the role of CDK2is in the first-line and early-stage setting remains undefined.

PROTAC-based degraders demonstrate the potential benefit of increased pathway inhibition with broader and more durable effects, including secondary disruption of CDK2 and cyclin E transcription as E2F targets. Yet, these agents have practical challenges due to poor oral bioavailability, degrader resistance through target mutation and E3 ligase downregulation and difficulties achieving consistent tumor tissue exposure.

A limitation of this Review is that most clinical claims are supported by preliminary reports and abstracts rather than peer-reviewed publications, as these agents remain in early-phase investigation. Accordingly, early efficacy and tolerability conclusions should be interpreted with caution. As mature trial data emerge, careful reassessment will be essential.

In the future, the most compelling remaining question may not be which CDK to target, but how to combine these therapies rationally and sequence them strategically based on the molecular landscape of each tumor. Several ongoing trials are investigating novel combinations of next-generation CDK inhibitors to define their role in the evolving HR^+^/HER2^−^ breast cancer treatment paradigm. A natural first step is to combine CDK4- or CDK2-selective inhibitors with oral SERDs or PROTAC-based ER degraders to achieve more complete suppression of both ER signaling and cell-cycle progression in the post-CDK4/6i setting. Combinations with PI3K inhibitors represent another important strategy, aimed at overcoming *PIK3CA* mutation–mediated resistance. Given the selectivity and improved safety profiles of these newer agents, such combinations may prove more tolerable than earlier regimens, and the results of these studies are eagerly anticipated.

Prospective biomarker-directed studies, in combination with longitudinal translational profiling of CDK complex dynamics under therapeutic stress, will be indispensable to continue stepping away from empirical combination testing toward precision cell-cycle therapy.

## Author contributions

GIS and AG conceived the Review. DS and JYK performed the literature review and wrote the initial draft. GIS and AG critically revised the manuscript for important intellectual content. JYK and AG prepared the figures. AG supervised the work. All authors approved the final version of the manuscript.

## Conflict of interest

GIS reports research funding from Merck KGaA/EMD-Serono, Artios, Eli Lilly, and Pfizer (all to Dana-Farber Cancer Institute); service on advisory boards for Merck KGaA/EMD-Serono, Circle Pharmaceuticals, Concarlo Therapeutics, Schrodinger, FoRx Therapeutics, and MycRx; and patents entitled, “Dosage regimen for sapacitabine and seliciclib,” and “Compositions and Methods for Predicting Response and Resistance to CDK4/6 inhibition.” AG reports research funding (exceeding $50,000 per year) from Cardiff Oncology (to Dana-Farber Cancer Institute); honoraria for consulting/advisory roles for Pfizer (exceeding $10,000 per year, but <$25,000), AstraZeneca (exceeding $10,000 per year, but <$25,000), Guardant Health, Araris, Eli Lilly, Incyclix, and Novartis; and travel grants from Eli Lilly, AstraZeneca, and Totus Medicine.

## Funding support

This work is the result of NIH funding, in whole or in part, and is subject to the NIH Public Access Policy. Through acceptance of this federal funding, the NIH has been given a right to make the work publicly available in PubMed Central.

- Elaine and Eduardo Saverin Foundation at Dana-Farber Cancer Institute grant to JYK.
- Harvard Cancer Consortium Specialized Program of Research Excellence (SPORE) in Breast Cancer (NIH/National Cancer Institute grant P50 CA168504) to GIS.
- Ludwig Center at Harvard to GIS.

## Figures and Tables

**Figure 1:**
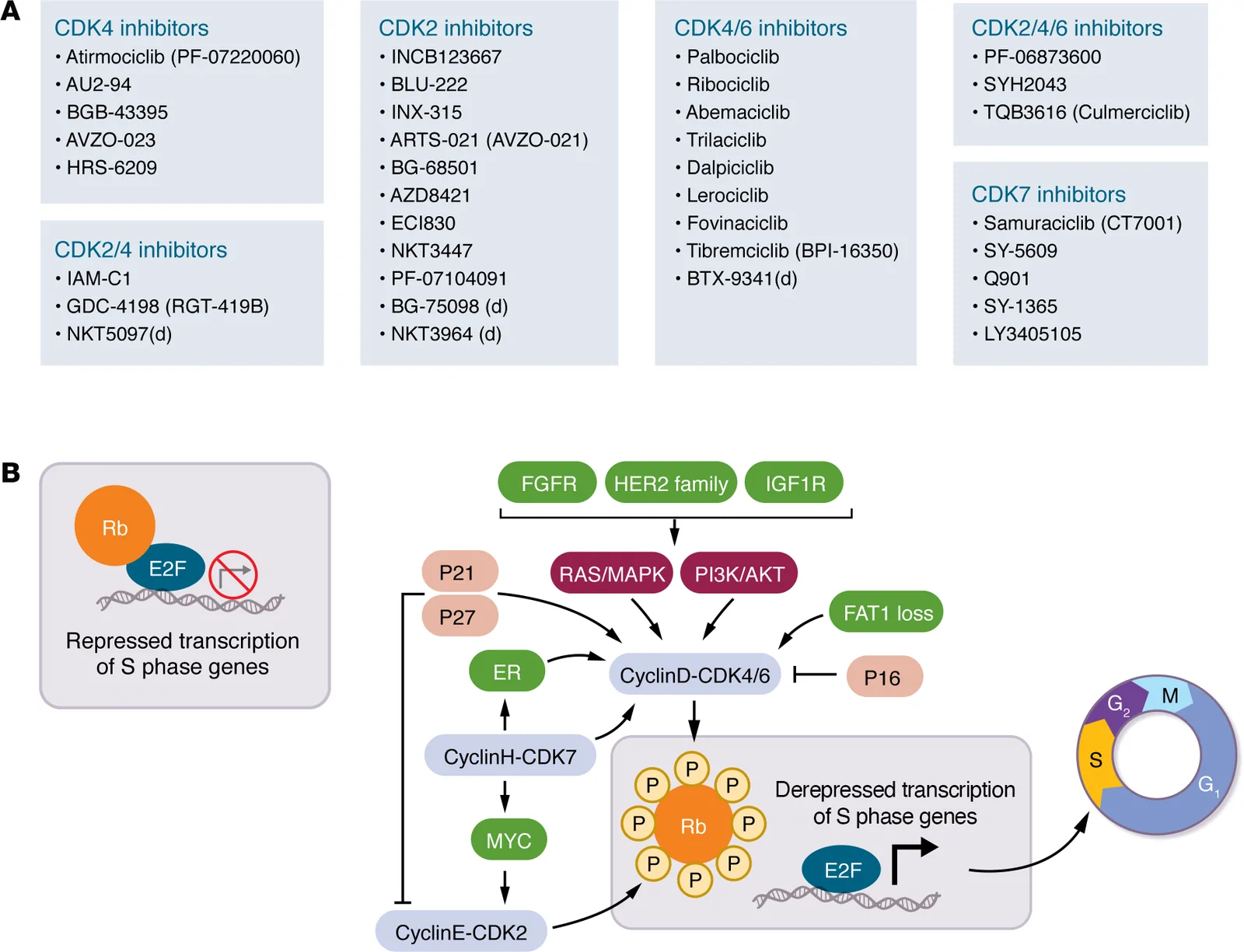
Overview of CDK inhibitors in development and role of the cyclin D–CDK4/6–Rb axis in cell-cycle progression in ER^+^ breast cancer. (**A**) CDK inhibitors are categorized based on their primary targets. d, degrader. (**B**) Upon mitogenic stimuli, activation of receptor tyrosine kinase and estrogen receptor (ER) signaling induce cyclin D1 expression and formation of cyclin D–CDK4/6 complexes in G_1_, leading to Rb hyperphosphorylation and liberation of E2F transcription factors for S phase gene expression. In ER^+^ breast cancer with resistance to CDK4/6is, these pathways are commonly dysregulated by CCND1 amplification, hyperactivation of upstream signaling pathways (including PI3K–AKT–mTOR, FGFR, and MAPK), and loss of FAT1, resulting in high cyclin D1 and CDK6 levels that enable persistent CDK4/6 activity. In addition, CDK7-driven MYC activation upregulates cyclin E and activates CDK2. These collectively drive sustained Rb hyperphosphorylation and uncontrolled G_1_/S progression.

**Table 2:**
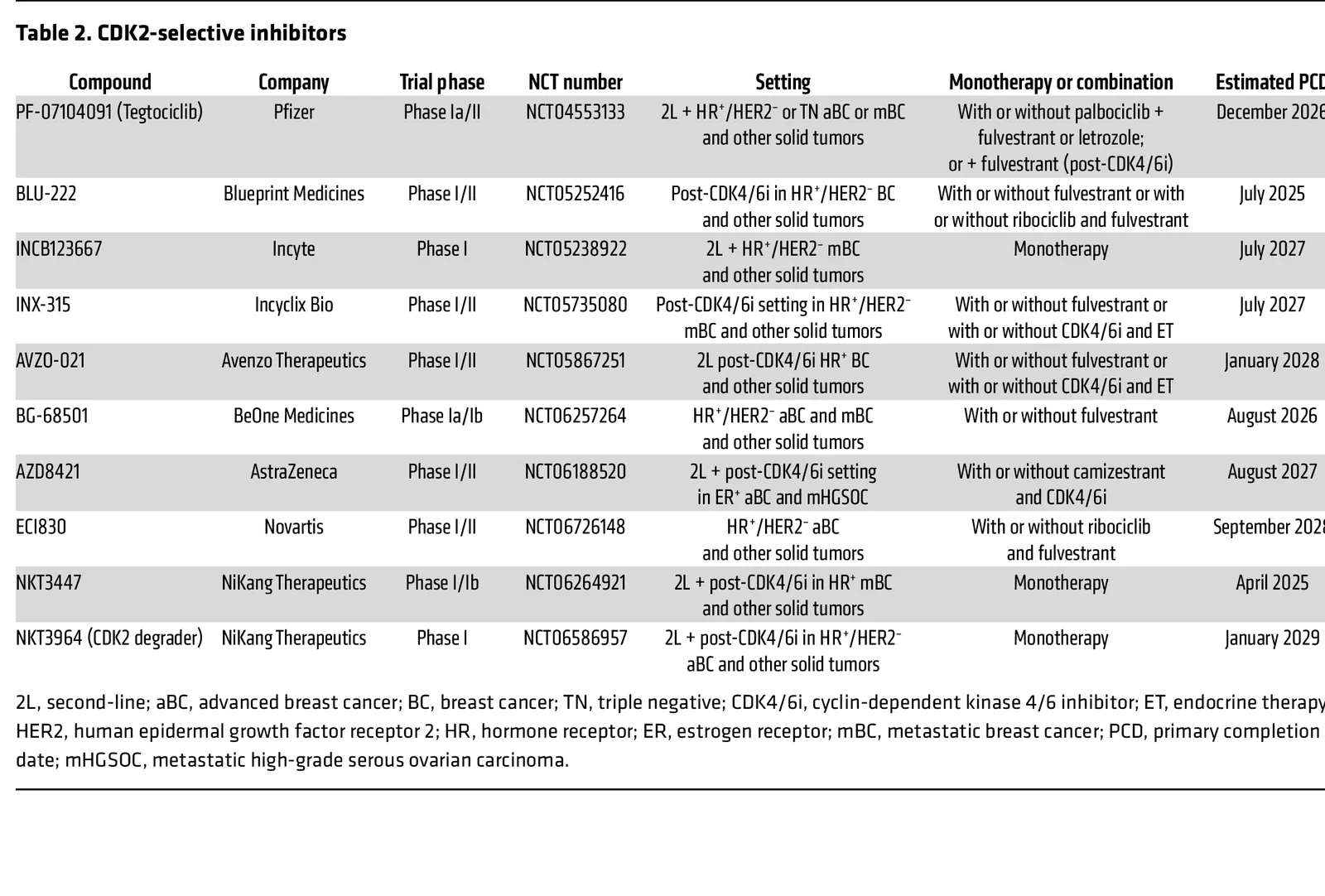
CDK2-selective inhibitors

**Table 3:**
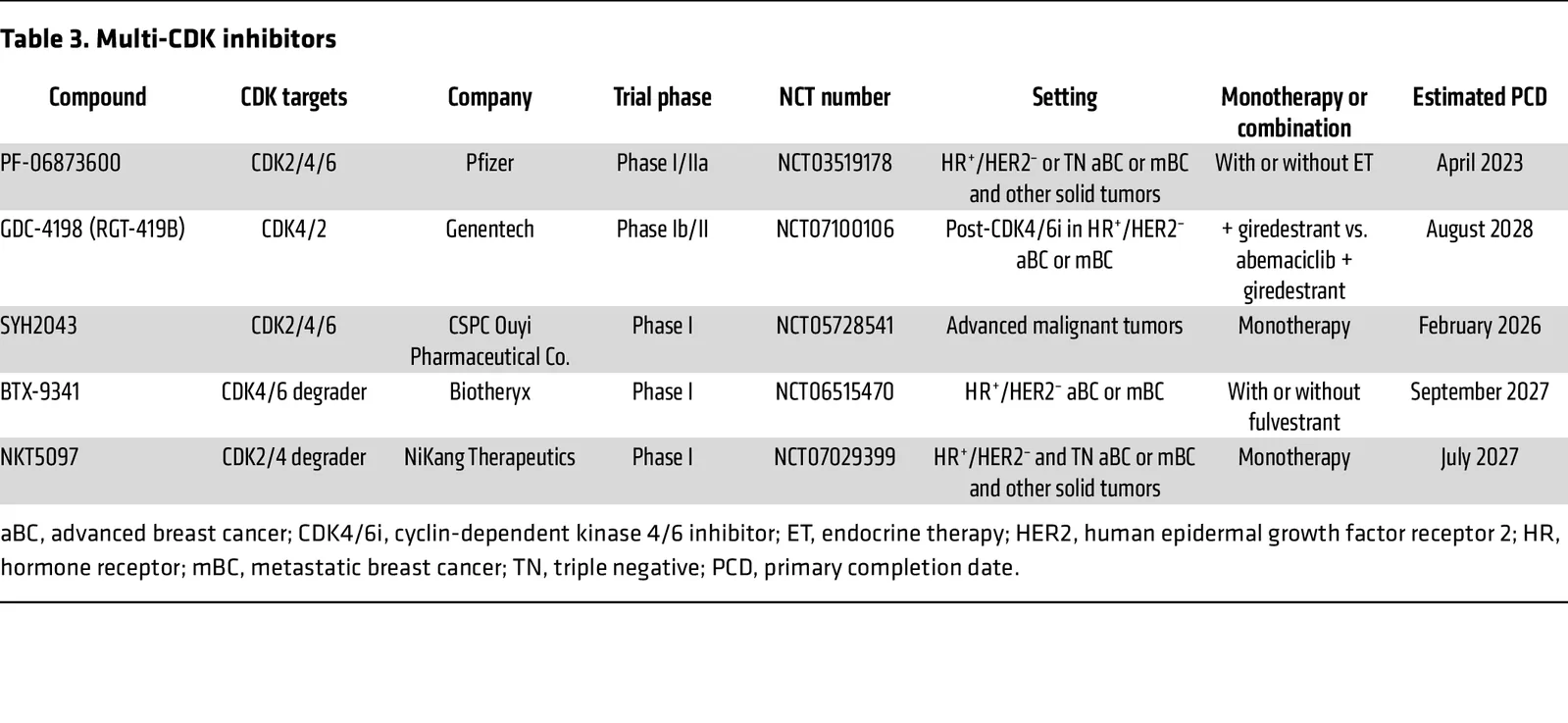
Multi-CDK inhibitors

**Table 1:**
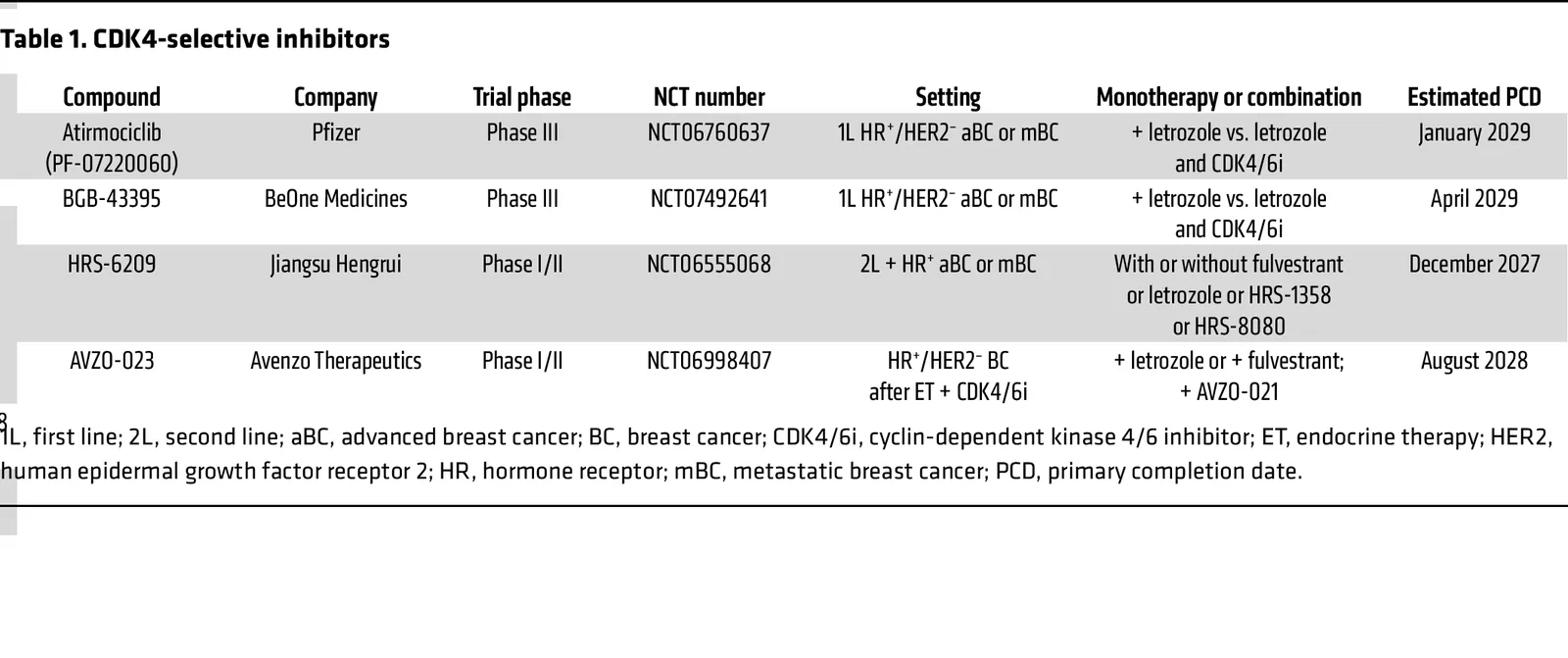
CDK4-selective inhibitors

## Version 1 10/15/2026

Electronic publication
